# Sedentarization and maternal childcare networks: role of risk, gender and demography

**DOI:** 10.1098/rstb.2021.0435

**Published:** 2023-01-16

**Authors:** Abigail E. Page, Andrea B. Migliano, Mark Dyble, Daniel Major-Smith, Sylvain Viguier, Anushé Hassan

**Affiliations:** ^1^ Department of Population Health, London School of Hygiene and Tropical Medicine, London WC1E 7HT, UK; ^2^ Department of Anthropology, University College London, London WC1E 6BT, UK; ^3^ Population Health Sciences, Bristol Medical School, University of Bristol, Bristol BS8 1TH, UK; ^4^ Graphcore, Lynton House, 7–12 Tavistock Square, London WC1H 9LT, UK; ^5^ Department of Anthropology, University of Zürich, Zurich 8006, Switzerland

**Keywords:** hunter–gatherers, allomothering, sedentarization, risk-buffering, grandmothering, gender-roles

## Abstract

Women cooperate over multiple domains and while research from western contexts portrays women's networks as limited in size and breadth, women receive help, particularly with childcare, from a diverse range of individuals (allomothers). Nonetheless, little exploration has occurred into why we see such diversity. Wide maternal childcare networks may be a consequence of a lack of resource accumulation in mobile hunter–gatherers—where instead households rely on risk-pooling in informal insurance networks. By contrast, when households settle and accumulate resources, they are able to *retain risk* by absorbing losses. Thus, the size and composition of mothers' childcare networks may depend on risk-buffering, as captured by mobile and settled households in the Agta, a Philippine foraging population with diverse lifestyles. Across 78 children, we find that childcare from grandmothers and sisters was higher in settled camps, while childcare from male kin was lower, offering little support for risk-buffering. Nonetheless, girls’ workloads were increased in settled camps while grandmothers had fewer dependent children, increasing their availability. These results point to gender-specific changes associated with shifting demographics as camps become larger and more settled. Evidently, women's social networks, rather than being constrained by biology, are responsive to the changing socioecological context.

This article is part of the theme issue ‘Cooperation among women: evolutionary and cross-cultural perspectives’.

## 1. Introduction

Women are frequently portrayed as having small, focused social networks due to differences in reproductive biology and childcare obligations (see [1,2]). However, across time and space, from anthropological, historical and demographic sources, it is evident that a wide range of individuals support women with children [3,4]. There is no one-size-fits-all supporter or ‘allomother’ (any individual other than the mother who invests in a child). Instead, those who are found to be important in terms of provisioning [5], childcare [6], child survivorship [7] and women's fertility outcomes [8] varies. While arguments of gender-based difference in social networks suggest this will be constant across socioecological contexts, it is apparent that women's social networks are flexible and respond to changes in subsistence and the environment [1,9–11]. Within a human behavioural ecology paradigm, such trends are expected as the fitness returns to cooperation—for both the mother and the allomother—will be dependent on the local socioecology [12,13]. Yet, little work has explored whether specific hypotheses can successfully predict diversity in allomaternal support. Here, we seek to examine if we can predict who provides childcare by exploring whether allomothering varies by different risk-buffering strategies present in mobile and settled Agta hunter–gatherers.

### (a) Risk-buffering

Risk is the probability of loss, losses that negatively impact fitness as they restrict organisms' ability to survive and reproduce. Consequently, adaptations are expected to evolve to limit these losses [14,15]. Research from diverse disciplines has pointed to the multitude of behavioural adaptations humans use to reduce risk [16–21]. Individuals are able to access a number of mechanisms depending on need and circumstance [22], and specific mechanisms cluster together and strengthen one another [15,23]. Some adaptations seek to *pool* risk by transferring it between households with short-term differences in wealth and resources (similar to insurance), while others seek to absorb or *retain* risk within the household with increased resources (i.e. stored food avoids starvation when harvests fail) [19]. Some types of adaptations actively conflict with one another, suggesting a trade-off between strategies [24,25]. Why households follow certain pathways in the first place is unclear, yet once a shift is made households appear progressively locked into specific adaptation clusters [21]. In hunter–gatherers, risk-buffering can be broadly grouped into either (i) *risk-pooling* alongside residential mobility [24,26,27] *or* (ii) *risk-retention* with sedentarization and resource accumulation. This theoretical divide between strategies allows us to develop predictions about the composition of maternal childcare networks.

### (b) Risk-pooling

Reciprocal cooperation has long been understood to reduce the risk of resource shortfalls in unpredictable and variable environments. Acting as a type of social insurance, cooperation can transfer risks—*risk-pooling*—between exposure units (individuals and/or households) at a small immediate cost, mitigating the severity of future losses [19,28,29]. In the case of mobile hunter–gatherers, risks are often associated with the daily variance in food returns in a stochastic environment [16,30], in addition to longer-term shortfalls associated with illness, accidents and disability that limit production and caring capabilities [31–33]. Shortages in one domain, such as food provisioning, impact other household domains (e.g. domestic work and/or childcare) as time, and thus energy, are finite [11,34]. For instance, mothers may increase foraging efforts if foraging returns are low due to sickness in the household and thus require substitutive childcare support [26]. There is a wealth of literature in human behavioural ecology about the trade-off between childcare and food production, particularly for women with young children due to the lack of compatibility between childcare and economic work [35,36].

Mothers with infants may reduce their investment in food production because of the constraints of breastfeeding and intensive needs of the infant [37–43], a pressure that reduces as children age [38]. A mother's reduction in food production results in shortfalls at the household level [44], which can be addressed by increased production of males [45], food sharing with other households [46,47] or by providing mothers with more childcare support. A number of studies have demonstrated that mothers who receive childcare support are able to increase the time spent in domestic and economic tasks [34,37,40,48–50]. For instance, in a detailed study of maternal time allocation in the Aka and Ngandu, Meehan [51] found that allomothers provided the majority of childcare when the mothers were busy in domestic food production tasks, targeting their investments. Based on this evidence then, it is reasonable to consider childcare as a *solution* to the viable foraging returns experienced by hunter–gatherers who are dependent on stochastic resources and face high rates of ill-health and disabilities. Food sharing can never be a perfect solution because there will be times when food sharing clusters are unable to address shortfalls if all have been unsuccessful [42]. In this case, childcare frees up the mother, allowing for increased food production, perhaps suggesting why we see such intra-individual variation in foraging returns [39], which are certainly dependent on the household's food acquisition at large [40].

By sharing resources when the household has these resources available (reducing the costs), the household can then reduce their losses later when this help is returned at a time of hardship (increasing the benefits). Therefore, reciprocal cooperation results in direct benefits (i.e. later-received help is more valuable [52]) and takes the form of food sharing [30], domestic labour [53] and childrearing [54], as all domains require time and energy to ensure the household needs are met, and cooperation easily moves between these different domains or currencies [36,37,55,56]. For this ‘risk-pooling’ to function, risk must be uncorrelated between exposure (i.e. households) units, allowing a redistribution of risk within the community [15,20,55]. Therefore, in mobile hunter–gatherer communities that face unpredictable losses, households are expected to have wide and diverse cooperative networks [26] to pool risk outside of the household to overcome the localized risks of foraging shortages, illness and accidents [30,33]. Previous research among the Agta [57] has demonstrated that the effect of reciprocity in childcare was strongest in less related individuals—suggesting their cooperation was dependent on direct benefits. Under a system of risk-pooling, we expect distant and non-kin to be more important allomothers in mobile camps—given the requirement for exposure units to be independent—and they would be more likely to be other mothers engaging in reciprocal childcare. Therefore, as we understand childcare to be a solution to unpredictable shortfalls, it follows that as households move away from foraging the requirement for reciprocity and diverse childcare networks diminishes as it does with food sharing.

### (c) Risk-retention

Material wealth (i.e. belongings) and food storage are a form of *risk-retention* as by increasing resources, households can absorb losses because they have a surplus [19]. Increases in resources then reduce the opportunity for household movement as increasing storage is associated with increasing permanence of settlements [20,25]. An increase in wealth and storage may further impact risk-pooling as well as mobility [20]*.* Given the abundance–shortage dynamic of reciprocal cooperation, wealthier individuals may find themselves overburdened by increased obligations to share while rarely requiring help themselves as risks are retained within the household [17,58–60] (but not consistently, see [61]). Individuals with more resources have less need for larger networks, withdrawing from them [59] as the direct benefits from reciprocal cooperation are reduced. Instead, more childcare may originate from household members (father and siblings) and grandparents, who receive indirect benefits of cooperation via inclusive fitness [62]. Schacht, Davis & Kramer [12] have suggested that among the Maya, economic development promotes the nuclearization of the household as more time is spent within the home, reducing relational wealth with wider social networks. There is good evidence that to optimize their entire multiplex social network, the same individuals will be cooperative partners in different domains, particularly if the relationships in some domains are important [63]. Therefore, we expect individuals to cooperate in childcare with the same households who provide food and other resources [64]. This dynamic is demonstrated in Starkweather *et al.*'s [56] investigation into the trade-offs between work and childcare in the Shodgar, in which women who traded together were more likely to provide childcare, increasing the range of helpers they had available to them and ensuring they received the assistance required. Therefore, settlement, and its associated increase in material wealth, is expected to result in smaller, more kin-focused networks as a result of wider changes to how households deal with risk. Specifically, we anticipate more childcare from close kin in wealthier, settled camps, and accordingly (beyond the father) these individuals are more likely to be pre- or post-reproductive individuals (siblings and grandmothers) who receive indirect benefits of cooperation.

### (d) Hypotheses and predictions

Previous research in the Agta has revealed that they have large childcare networks, and while mothers provide a significant proportion of childcare, this decreases with children's age as they are increasingly involved in sibling and playgroup care. Grandparents represent a very small proportion of allomaternal care due to few being alive and present, but their care, like that of playgroups, actively substitutes a mother's investment, reducing her workload [34]. Here, using the same in-depth focal follow data on 78 Agta children (aged 0–5.9 years), we test if these maternal childcare networks are related to settlement, exploring whether we can predict who are important allomothers based on different risk-buffering strategies. The Agta from Palanan, Philippines, follow a mixed subsistence strategy that involves differing involvement in foraging, wage labour and cultivation [65]. As a result, they demonstrate significant variation in mobility and wealth accumulation [66], representing opposing risk-buffering strategies—risk-pooling in mobile camps with little material wealth and food storage and risk-retention in settled camps with greater accumulation of resources—which we expect to influence a mother's childcare networks. Specifically, we predict that (1) in mobile camps childcare will be reflective of *risk-pooling strategy* as distant kin and non-kin (i.e. networks comprising independent units) will provide more childcare than in settled camps where (2) more childcare will originate from the household (father and siblings) and grandparents (particularly grandmothers), representing a *risk-retention strategy*. Therefore, we expect a mother's childcare networks to be smaller in settled camps and larger in mobile camps (prediction (3)). Further we predicted that (4) in mobile camps other mothers (i.e. reproductively active women) will be more involved in childcare, based on direct benefits received from reciprocity, as compared to settled camps where (5) more childcare will originate from post- and pre-reproductive allomothers, based on indirect fitness returns. By exploring what predicts diversity in allomothering, we hope to gain insight into how allomothering functions, and how women's social networks are adaptive to major livelihood transitions, rather than being fixed by reproductive constraints [1].

## 2. Methods

### (a) The Agta

There are around 1000 Agta living in the Palanan municipality of northeastern Luzon, Philippines. Riverine and marine spearfishing provides their primary source of animal protein, supplemented by hunting and gathering, as well as low-intensity cultivation, wage labour and trade [65,67]. This variation in subsistence is mirrored in the types of camps the Agta live in, as some camps are *settled* with permanent structures, are larger and have some form of infrastructure like a drinking well or church. Other camps are *mobile*, comprising temporary shelters, are smaller in size and the people who reside in these camps change frequently [66]. As previously demonstrated [66], camp type correlates with household wealth and food storage; therefore, we use camp type (mobile versus settled, based on definitions above) as a proxy for the different risk-buffering strategies. Further ethnographic detail can be found in the electronic supplementary material, methods.

### (b) Data collection

Data collection occurred over two field seasons from April to June 2013 and February to October 2014. In the first season, we censused 915 Agta individuals (54.7% of which were men) across 20 camps, capturing the majority of the population. Following relative ageing protocols [68], accurate ages were established for all individuals post-data collection. Relatedness was established from reproductive histories (with mothers) and household genealogies (involving both mothers and fathers; see electronic supplementary material, methods). In the second season, we stayed approximately 10–14 days in 10 camps to conduct focal follows with 78 children: 34 children aged 0–1.9 years and 44 children aged 2–5.9 years (electronic supplementary material, table S3). Two researchers (A.E.P and S.V.) observed each focal child for a 9 h period broken into three 4 h intervals (6 : 00–10 : 00, 10 : 00–14 : 00 and 14 : 00–18 : 00, with 15 min breaks each hour) on non-consecutive days. During observations, researchers recorded the activities of the focal child every 20 s and who came within 3 m of the child and engaged in low- or high-investment forms of childcare. Low-investment activities include touching, proximity watching, supervising, being in a playgroup with a child or talking to a child (also referred to in the literature as indirect childcare). High-investment activities included feeding, cleaning, holding or carrying, playing or otherwise actively engaging with the focal child (following [69]). Once accurate ages had been produced, allomothers were defined as all individuals aged above 6 years of age. Further information can be found in the electronic supplementary material, methods and full protocols are published in [34]. It is important to note that our methodology differs from similar studies as we recorded proximity interactions at 3 m (as compared to only holding, carrying, touching or arms-length (reviewed in [70])). We focused specifically on a wider range of low-investment behaviours because they constitute an important form of childcare [57,71] and our sample includes children up to the age of 6, whose care involves less high-investment engagement and more passive supervision. In comparison, previous studies often include focal children up to the age of 3 or 4 years (reviewed in [70]), another consideration in terms of cross-cultural comparisons.

We (M.D., D.M-S. and A.E.P.) also conducted daily camp scans starting between 6 : 30 and 9 : 30 and then recorded three more scans at 3 h intervals (see [65]). In each scan, we recorded the current activity of every camp member. When individuals were out of camp, we asked those in camp what the absent individuals were doing and verified this when the individual returned. Time allocation categories of interest here included domestic tasks and out-of-camp activities (see electronic supplementary material, table S2). During household interviews, we also collected data on household wealth, food stored (the amount of rice stored in the household measured in kg, range 0–14 kg) and house permeance. Household wealth is a continuous variable based on a weighted count of the number of objects (see electronic supplementary material, table S1 for a breakdown) within the household (range 0.18–5.5). Household permeance was a 0–1 scoring system to quantify the type of houses individuals lived in; simple lean-tos were allocated 0, while permanent cement constructions were allocated 1 (range 0–0.88).

### (c) Data analysis

#### (i) Maternal childcare networks

To look at the characteristics of mothers' childcare networks, we detailed all the allomothers with whom children interacted (at least once during focal follows) from the mother's perspective. This created an unweighted network (i.e. a link between two individuals) broken into four key kin categories: distant kin (mother's brother, sisters, nieces and nephews, cousins and aunts and uncles), household (mother's partner and children) and parents/parents-in-law and grandparents (if surviving) and non-kin (*r* < 0.0325). Using the count of individuals within mothers' childcare networks, we explored whether the sizes (separated into low-investment and high-investment networks) of mothers’ childcare networks were predicted by camp settlement using *t*-tests.

#### (ii) Kinship and reproductive status models

We ran negative binominal (due to overdispersion) mixed-effect models in R v.4.0.3 [72] using the *glmmTMB* package [73] to predict the number of interactions between a child and allomother by their kinship relationship, reproductive status and sex, and whether they lived in a settled or mobile camp. Due to the structure of data collection, allomothers are only entered into the models if they were observed investing in a child at least once during the focal follows. Individuals present in camp, but who were never witnessed investing in a child were unable to be included in the models as we did not keep a daily record on who was present in camp to provide this care. Two sets of models were run: (i) kinship models—testing if household care was more common in settled camps, and (ii) reproductive and sex status models—testing if more care originated from post- and pre-reproductive individuals in settled camps. In the following analyses, childcare by mothers and allomothers has been categorized into low-investment (passive engagement) or high-investment childcare (active engagement). Therefore, each of the two model sets had two outcome variables: (i) sum of high-investment interactions and (ii) sum of low-investment interactions. This produced a total of four pre-planned models to test our hypothesis, for which full results are presented in the electronic supplementary material results alongside model diagnostics conducted in *DHARMa* [74].

While the majority of children were watched for a total of 1080 observational periods, there was some variability. As a result, the models were offset by the total number of interaction periods. All interactions between mothers and children were removed from the dataset. The unit of analysis in the model was the dyadic relationship (*n* = 1,522) between a child (*n* = 78) and allomother (*n* = 362). Random effects captured clustering at the camp (*n* = 10) level, as well as the repeated observations from children and alloparents in different dyads. We originally intended to include household as a random effect; however, we encountered convergence issues. The random effect variance attributed to the child household level was nil, thus its removal had no impact on the model.

We used directed acyclic graphs to illustrate the hypothesized causal relationships between variables and to identify which confounders to adjust for (see electronic supplementary material, figures S4 and S5) using the *dagitty* package [75]. In the kinship analysis, we controlled for child age, whether the camp was coastal or inland and how many siblings the child had. In the reproductive and sex status models, we controlled for the same variables in addition to kinship (captured continuously with *r,* the coefficient of relatedness) and the sex of the child. In the kinship models, relatedness was from the child's perspective and expressed as a seven-level categorical variable: father, brother, sister, grandmother, grandfather, distant kin (*r* ≥ 0.03125 and *r* ≤ 0.25, but excluding grandparents as named above) and non-kin (*r* < 0.0325). The reproductive and sex status models were expressed as a six-level categorical variable: boy, girl (aged 6–14.9 years), man, woman (aged 15–45 years) and post-reproductive man and post-reproductive woman (aged 45 years or more). All models were run with an interaction between the predictor variable and camp status. Camps were defined as either settled (*n*_camp_ = 7, *n*_children_ = 60) or mobile (*n*_camp_ = 3, *n*_children_ = 18); the difference in sample size between the two was a product of visiting more settled camps, which had more inhabitants on average (settled = 56 ± 30 versus mobile = 38 ± 11).

#### (iii) *Post hoc* models: grandmaternal care load and gendered activities

To further explore our results from the mixed-effect models, we conducted two *post hoc* analyses. First, to explore grandmaternal care load, we ran a Poisson generalized linear model to test whether settlement predicted the number of biological children aged under 11 that grandmothers currently had (as reported in birth histories). This model controlled for grandmaternal age (*n* = 24). The second analysis explored the relationship between gender and settlement for juveniles. Here, using camp scan data of daily activities, we ran a mixed-effects model (clustered by camp) to test whether an interaction between age and sex of the child (aged 6 to 15.9 years) predicted the proportion of activities spent in either (i) domestic tasks or (ii) out-of-camp activities. These models controlled for child age and whether or not the camp was coastal as daily activities were influenced by whether the camp was inland or on the coast (*n* = 108). The code and the data used in these analyses can be found on Open Science Framework (OSF; https://osf.io/5cghy/?view_only=c2cb9f7595ac41dbb55a430091560584).

## 3. Results

Summary statistics, separated by mobile and settled camps, are presented in electronic supplementary material, table S1. The focal children's age (*t*_24_ = −0.236, *p* = 0.816) and sex (*c*^2^ = 0.189, *p* = 0.663) did not differ significantly by camp type, nor did number of siblings (*t*_24_ = −1.488, *p* = 0.146). However, settled camps were significantly more likely to be found on the coast than inland (*c*^2^ = 4.622, *p* = 0.032). As expected, household permanence (*t*_24_ = −6.763, *p* < 0.001) and wealth (*t*_24_ = −3.271, *p* = 0.002) were higher in settled camps. While the proportion of activities spent in hunting–gathering was higher (mother *t*_24_ = 1.067, *p* = 0.296; father *t*_24_ = 0.433, *p* = 0.669) and food storage (*t*_29_ = −0.245, *p* = 0.808) lower in mobile camps these were not strong results, highlighting less difference between mobile and settled households than expected. The lack of strength between these associations for food storage and hunter–gathering, contrary to previous findings [66], may reflect the small number of households in mobile camps.

### (a) Maternal childcare networks

**Figure 1.**
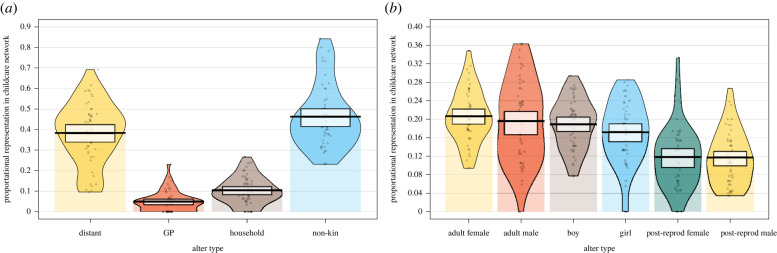
Distribution of unweighted ties in mothers' childcare networks (*n* = 50) separated by (*a*) kinship and age and (*b*) sex category from the mother's perspective. Here, distant kin includes the mother's brothers, sisters, nieces and nephews, cousins and aunts and uncles, while household includes their partner and children. Grandparents (GP) includes their parents, parents-in-law and their own grandparents, if surviving. (Online version in colour.)

Our sample comprises 50 mothers of 78 children whose low-investment childcare networks, on average, comprised 25 allomothers (s.d. = 11.67), ranging from 10 to 65 separate helpers. The high-investment childcare networks were predictably smaller, with on average 9.9 allomothers (s.d. = 3.8, min = 2, max = 19). Non-kin made up a large proportion of the unweighted links in the childcare network (i.e. if at least one instance of allomothering occurred), accounting for 46.6% (95% CI = 41.40–50.31%) of ties, household members (partners and children) consisted of 10.5% of the network (95% CI 8.3–12.1%) followed by parents and grandparents (including in-laws) (5.0%, 95% CI 3.5–6.3%, figure 1*a*). Mothers' childcare networks were predominantly comprised other reproductively active females (20.7%, 95% CI 18.8–22.1%) and males (19.6%, 95% CI 16.9–22.2%), while post-reproductive individuals had the lowest representation (females = 11.9%, 95% CI 9.9%−13.8% and males = 11.7%, 95% CI 10.0–13.3%, figure 1*b*).

We ran two *t*-tests to test if mothers in mobile camps had larger childcare networks than mothers in settled camps. These tests indicated results that were inconsistent with our predictions. For low-investment childcare, mothers in settled camps had, on average, significantly larger childcare networks than mobile mothers: 25.4 versus 18.8 (95% CI [−11.2, −2.0.], *p* = 0.006, *t*_47_ = −2.873). The size of the childcare network based on high-investment activities showed no such difference: 10.2 versus 9.1 (95% CI [−3.2, 1.0], *p* = 0.287, *t*_47_ = −1.079).

### (b) Kinship, settlement and childcare

**Figure 2.**
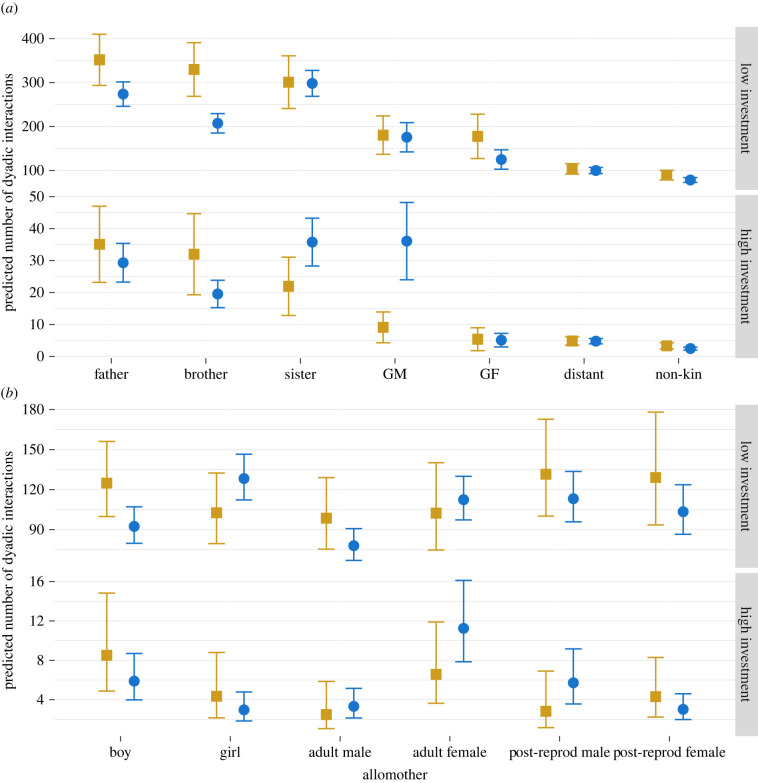
Predicted probabilities for the percentage of dyadic interactions separated into low- (top facet) and high-investment (bottom facet) and camp type (settled, blue circles; mobile, yellow squares) for allomothers separated by either (*a*) kinship or (*b*) sex and reproductive status. Error bars are 95% confidence intervals, *n* = 1522. GM, grandmothers; GF, grandfathers; distant, distant kin. Boy and girls are juveniles aged 6–16 years, adults are aged 17 to 44.9 years and post-reproductive adults are aged 45 years plus. Please note the different y-axis scales. (Online version in colour.)

Children on average received 122.5 instances of low-investment (s.d. = 147.4, max = 960.0) and 10.3 instances of high-investment (s.d. = 30.8, min = 0.0, max = 440.0) childcare from any given allomother. When controlling for a child's age, number of siblings and whether the camp was inland or coastal, and consistent with our predictions, children's grandmothers provided significantly more high-investment childcare (figure 2) in settled camps as compared to mobile camps (rate ratio (RR) = 3.960, 95% CI [1.168, 13.429], *p* = 0.027). Grandmothers in settled camps were predicted to provide 36.1 instances of high-investment childcare, compared to 9.1 in mobile camps. Children's sisters followed a similar trend, providing more high-investment childcare in settled camps (RR = 1.630, 95% CI [0.663, 4.008], *p* = 0.287); however, the strength of evidence for this was weaker. No trends were apparent for grandmothers and sisters in the low-investment models. Contrary to our predictions, the children's fathers and brothers were not found to provide more childcare in settled camps and, in fact, were found to be 16.5% and 38.8% less likely, respectively, to provide further care in settled than in mobile camps (but not significantly so).

The low-investment models repeated the trend of male relatives providing less childcare in settled camps as compared to mobile camps, contrary to our predictions. Brothers were predicted to provide 207.3 instances of low-investment childcare in settled camps, compared to 329.7 instances in mobile camps (RR = 0.629, 95% CI [0.414, 0.956], *p* = 0.030), an effect that was observed, but with less strength, in fathers (RR = 0.778, 95% CI [0.531, 1.140], *p* = 0.198) and grandfathers (RR = 0.704, 95% CI [0.365, 1.355], *p* = 0.293). Furthermore, we witnessed no trends of receiving less childcare from distant and non-kin in settled camps, as predicted.

### (c) Reproductive status, settlement and childcare

The reproductive and sex status models highlighted similar gendered differences, offering little support for our hypotheses. While little variance was apparent in the high-investment models, the low-investment models showed that settled boys were predicted to provide 92.4 instances of childcare, which was significantly less than 124.8 instances in mobile camps (RR = 0.740, 95% CI [0.569, 0.962], *p* = 0.025). In line with the kinship models, adult males were less engaged in childcare in settled camps; however, the confidence intervals overlap (RR = 0.791, 95% CI [0.584, 1.070], *p* = 0.128). There was no evidence that reproductively active allomothers provided more care in mobile camps, or pre- or post-reproductive individuals provided more in settled camps, as predicted.

### (d) Gender, settlement and daily activities

**Figure 3.**
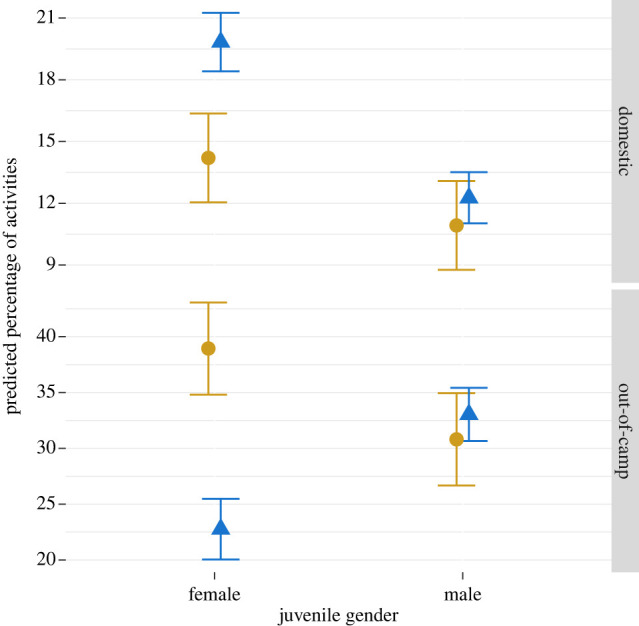
Predicted probabilities for the percentage of activities spent in domestic tasks (top facet) and out-of-camp activities (bottom facet), dependent on camp type (mobile, yellow circles; settled, blue triangles) and the sex of the individuals on the *x*-axis. Error bars are 95% confidence intervals, *n* = 108 of children aged 6–16 years. Please note the different *y*-axis scales. (Online version in colour.)

We conducted a follow-up analysis on the relationship between gender, daily activities and camp type for juveniles aged 6–16 years. These models highlighted that gendered division of labour in the juvenile period differed by camp type, matching the childcare models (figure 3). In mobile camps, the percentages of domestic tasks (out of all daily activities) that boys and girls conducted were broadly similar (boys = 10.9%, 95% CI [6.6, 15.2]; girls = 9.9%, 95% CI [9.9, 18.5], *p* = 0.277). By contrast, in settled camps girls spent 19.8% (95% CI [17.01, 22.64]) of activities in domestic tasks, while boys remained relatively unchanged at 12.2% (95% CI [9.8, 14.7]). Consequently, girls spent significantly more activities in domestic tasks in settled than in mobile camps (*β* = 5.631, 95% CI [0.633, 10.629], *p* = 0.027). Out-of-camp activities highlighted a similar trend. In settled compared to mobile camps, girls were less frequently out of camp (*β* = −16.179, 95% CI [−25.784, −6.610], *p* = 0.001), as well as compared to boys in settled camps (*β* = −10.287, 95% CI [−17.627, −2.929], *p* = 0.006). There was no sex difference in mobile camps.

### (e) Grandmothers, settlement and number of dependents

While the sample of grandmothers was small (*n* = 24), given their absence in the population at large [34], we nonetheless find significant differences between the number of dependents (children aged 11 years or less) that grandmothers have in settled camps (average of 1.0 dependent, s.d. = 1.86) as opposed to mobile camps (2.3 dependents, s.d. = 1.66, *β* = −1.059, 95% CI [−1.541, −0.351], *p* = 0.005).

## 4. Discussion

Contrary to our predictions, we find no evidence that settlement in the Agta was associated with alternative risk-buffering strategies in mothers' childcare networks. Specifically, while we find that receiving childcare in settled camps (a proxy for risk-retention) was more likely from some closer family members, grandmothers and sisters, it was less likely from others—brothers and fathers. Maternal childcare networks did not become narrower, nor were they more nuclearized [12], with decreased mobility and increased wealth. This suggests that increasing wealth and settlement are not like-for-like substitutes for risk-pooling in this context [61], or at least, this is not captured in childcare networks.

Discussing null results is always challenging as there may be a number of ecological, theoretical and methodological reasons why our predictions were not supported. Ecologically, it may be that (fishing-focused) food production is not as incompatible with childcare in the Agta as we have assumed—as compatibility depends on the types of economic tasks (see [56] for discussion)—reducing the need to use childcare as a solution to food shortfalls. Certainly, in the coastal population, women often engaged in marine foraging in the inter-tidal zones, collecting shrimps, crabs, mollusc and octopus. Such tasks are more compatible with childcare than other forms of fishing in the population, and young children were often brought along [76]. Nonetheless, Agta women did reduce their workload [65] and calories produced [42] when they had young, dependent children, suggestive of a trade-off. Another consideration is that whether mothers reduce or increase their childcare in the face of allomaternal investments is dependent on the environment, the skills required for childcare and efficient food production and the risk to children when mothers were absent [40]. Therefore, childcare may not act as a solution to variability in food production if mothers optimize by increasing childcare. This consideration, however, seems unlikely because we have previously demonstrated that allomaternal childcare substitutes maternal care, increasing mothers' time in domestic and economic tasks [34] as documented in other populations [37,48–50].

Turning to the theoretical issues, another consideration is that cooperative childcare may not function to risk-pool in the Agta but reflect alternative motivations for cooperation. Here, we have not explored the gains acquired from cooperation—be those indirect benefits associated with relatedness, direct benefits associated with reciprocity or increased returns associated with mutualist turn-taking. For instance, rather than one woman helping another when her household has a surplus and the other a shortage (as predicted by risk-pooling), two households might take-turns in crèching their children, leaving one mother to watch the children so the other can more efficiently forage. Jaeggi *et al*. [55] found such trends in the Tsmaine, in which childcare (unlike other domains like food sharing) was exchanged in-kind, suggestive of turn-taking. In addition, it may not be that risk strategy varies as a function of sedentarization (as predicted), but rather childcare networks respond to environmental risks associated with sedentism. Levels of infectious disease morbidity and mortality are higher in settled camps [66], which may foster wider cooperative networks beyond the household and close kin if associated sickness associated losses cannot be absorbed within the household [32,77]. In the face of high levels of infectious disease, mothers with diverse and independent networks may receive help when most needed [26].

Finally, in terms of methodological issues, it may be that our study is limited by the consideration of only one cooperative domain. Specifically, reciprocal cooperation has been documented to function across different domains, or currencies to ensure that households are buffered from shortfalls [55]. As we have not included in our analysis cooperation in food sharing, production and domestic labour we are ignoring the links between the layers in women's multiplex social networks. As argued by Atkisson and colleagues [63], this means we risk drawing the wrong conclusions because the cooperation that occurs in one domain can structure another [56]. Relatedly, a further issue is that the distinction between mobile and settled camps is not great enough in our sample. Certainly, while there are some differences between settled and mobile camps, the level of wealth accumulation is still relatively minor and residence in settled camps does not preclude residential mobility [66]. Therefore, our measures of risk-buffering strategy may not be adequate as the process of sedentarization—which is frequently nonlinear—may not be far enough along. Further research is necessary to explore these considerations and questions if we are to better understand these null findings.

Overall, mothers in this study had access to diverse social networks and received childcare help from a wide number of related and unrelated individuals, echoing previous findings in the Agta [26,78] and elsewhere [2,79–82]. The wide range of kin and non-kin who provide childcare has now been documented in a number of small-scale societies [43,49,83] across different subsistence types. This suggests that's Hrdy's [84] assertion was well founded, that mothers require large, flexible networks to ensure they receive the help required. Therefore, in line with other contributions to this special issue, women are not constrained to small, dyadic networks [1,10,85]. Interestingly, the low-investment networks were large, comprising of, on average, 25 different allomothers over the course of the 3 days of observations. Similar studies have found much smaller network sizes, averaging around 11 or 12 allomothers [43,70,83]. This is likely a function of our focus on more ‘passive’ forms of childcare, in the form of proximity watching, playgroups or supervision at a distance, as well as the inclusion of older children aged up to 6 years, who interact with more people in less labour-intensive ways [34]. In line with this, the Agta's high-investment networks are consistent with the cross-cultural literature, with an average of 10 allomothers. It is of particular note, in line with the relative absence of grandmothers in the population at large [34], that post-reproductive individuals were least represented in mothers' childcare networks. Childcare networks mainly comprised pre-reproductive juveniles and reproductive-aged adults, contrary to cooperative breeding models focused on the post-reproductive lifespan [86,87]. However, we saw no variation in the reproductive status of allomothers by camp type as predicted, which suggests that the relative importance of direct (reciprocity) and indirect (kin selection) benefits did not vary by sedentism. Nonetheless, the role of both grandmothers and siblings appeared to be particularly responsive to degree of settlement, a potential function of demographic strategies influencing who mothers have access to as allomothers.

Camps did not vary in terms of levels of relatedness (perhaps related to the multi-level nature of Agta camps, in which more related households form their own clusters in camp [42]), but there appeared to be changes in grandmothering trends. Our strongest results demonstrated that levels of grandmaternal care appeared dependent on sedentarization. Previous research shows that who cares, or who is available to care, varies by market integration [88], degree of market access [12] and level of urbanization [89]. The extent to which certain relatives help (or not) can be attributed to different levels of competition and/or cooperation within groups; co-resident or nearby kin can be both competitors as well as provisioners, with chances of conflict more likely when local resources are limited [90–95]. For instance, among the horticulturalist Pimbwe population in Tanzania, Hadley [58] found that wealthier women with larger kin networks suffered excessive demands on their resources, with negative effects on their children's nutritional status. In the case of the Agta, as settled camps had increased levels of household wealth and food storage, local resource competition may have been reduced due to greater resource availability. Furthermore, grandmothers in settled camps had fewer dependents, which reduced their own caregiving needs and the degree of reproductive conflict [34,96,97], potentially increasing ‘grand-allomothering’. Previous research has demonstrated that previously mobile Agta women residing in sedentarized camps experienced both higher childhood mortality rates (associated with settled camps) and lower fertility (associated with residential mobility), reducing the number of surviving children [66]. Therefore, the demographic trends associated with the health costs of sedentarization may underpin alterations to allomothering. Research among pre-industrial Finnish populations similarly highlighted the importance of demographics, specifically that older grandmothers were negatively associated with grandchild survival as their ill-health increased, reducing their ability to help [98]. These findings underline the importance of considering both the costs and benefits of cooperation, and how demographic transitions impact maternal networks [13,88].

Beyond grandmothering, clear gender-based changes in childcare were apparent in our data. Specifically, high-investment care from sisters appeared increased in settled camps and low-investment care from brothers decreased. As a result, while sisters' and brothers’ childcare did not significantly differ in mobile communities, it did in settled ones. In line with these findings, our analysis of children's time allocation demonstrated relatively little sex differentiation in mobile camps, while in settled camps girls conducted significantly more domestic tasks and were out-of-camp significantly less. Similar findings are apparent throughout the hunter–gatherer literature [99,100]. For instance, in settled !Kung communities children's involvement in unskilled tasks increased, as did the gendered division of labour. Girls were more involved in childcare and household tasks, while boys spent more time away from camp, herding or seeking water or wood [101,102]. Such findings are in line with a wide range of cross-cultural research [103,104].

Why gender roles shift so significantly may be a function of the changing nature of household economy. Agricultural food production is commonly associated with an increasing workload [65], which frequently falls into the women's domain [102,105] (given compatibility with childrearing, though the sex division of labour is more fluid in foraging communities [106]). These tasks are also increasingly possible for children with less skill and strength to complete [96,100,107–109], increasing children's workload [101,110]. Thus, increasing gender segregation occurs, with downstream effects for children's work. For instance, in a cross-cultural analysis Lew-Levy *et al*. [111] found that sex differences in children's work increased in societies with stricter sexual divisions of labour. Cross-culturally, girls tend to prefer more face-to-face time with adults, particularly, women, than boys (who more often assort with male peers) [96,102,112]. Consequently, Draper [102] called girls ‘pre-adapted’ to take on these increasing domestic and childcare duties of women because they are proximate and ready targets to be assigned household tasks by mothers [100,104,112] who have more to do and are keen to reduce their workload [96]. In comparison, boys are more frequently out-of-camp and spend less time with adults (and infants) and more time with male peers, giving them greater freedom from adults and domestic work [100–102,112].

These sex-based changes to mobility and assortment may further be related to demographic changes that alter the nature of playgroups. Mixed-age and mixed-sex playgroups are a ubiquitous feature of mobile hunter–gathering populations [80,113,114]. Arguably, they are the consequence of small camp sizes that cannot support significant clustering by age and sex [101,115,116] despite children preferring to play with similar-aged and same-sex peers because of shared interests and behaviours [117]. In particular, cross-culturally boys are much more frequently engaged in physical and rough-and-tumble play than girls [110,112]. In mixed-age, mixed-sex playgroups these interests are modified and less gender-specific and competitive play occurs [113,118], supporting the integration of younger children [34] and limiting the distance travelled from camp. Therefore, in mobile camps, there are few gender differences in childcare within playgroups, and in spatial mobility in and around camp. However, as camp sizes get larger with settlement (we note that table 1 shows no significant difference in mean camp sizes between the two camp types in this sample, however, mobile camps are smaller on average, and the range is much larger in settled camps. Further, in a more complete sample of Agta camps, settlement is clearly associated with camp size [66]), children increasingly cluster into same-sex, similar-aged groups as the availability of playmates increases [116]. In settled camps, it was common to witness boy-only playgroups, without the toddlers, which would roam further from camp and engage in riskier activities [119]. By contrast, given the reasons above, girls remained in camp and thus were more readily engaged in childcare and domestic tasks. These findings underscore that mothers' childcare networks not only change due to maternal interests or strategies (e.g. risk-buffering), but due to exogenous factors, such as the demographic composition of camps. As with the grandmother-specific findings, this suggests that we must pay attention to population-specific demographic composition and trends.

**Table 1.** Descriptives of key camp variables separated by camp settlement type. *p*-values are reported based on ANOVA or chi-squared tests dependent on data type. Sample sizes are consistent across variables, expect for household wealth (*n* = 71, 18 mobile and 53 settled), camp size (*n* = 10, 3 mobile and 7 settled) and measures of relatedness (*n* = 74, 17 mobile and 57 settled).

	mobile (*n* = 18)	settled (*n* = 60)	total (*n* = 78)	*p*
age of child				0.796
mean (s.d.)	2.26 (1.81)	2.37 (1.52)	2.35 (1.58)	
median (min, max)	1.62 (0.08, 5.75)	2.21 (0.08, 5.92)	2.08 (0.08, 5.92)	
sex of child				0.664
male	11 (61.1%)	40 (66.7%)	51 (65.4%)	
female	7 (38.9%)	20 (33.3%)	27 (34.6%)	
mother hunter–gatherer				0.242
mean (s.d.)	0.50 (0.48)	0.37 (0.40)	0.40 (0.42)	
median (min, max)	0.67 (0.00, 1.00)	0.23 (0.00, 1.00)	0.25 (0.00, 1.00)	
father hunter–gatherer				0.637
mean (s.d.)	0.72 (0.37)	0.68 (0.32)	0.69 (0.33)	
median (min, max)	0.85 (0.00, 1.00)	0.75 (0.00, 1.00)	0.79 (0.00, 1.00)	
house storage				0.812
mean (s.d.)	1.72 (3.12)	1.93 (3.29)	1.88 (3.23)	
median (min, max)	0.50 (0.00, 10.00)	1.00 (0.00, 14.00)	1.00 (0.00, 14.00)	
house wealth				0.022
mean (s.d.)	1.58 (0.59)	2.30 (1.26)	2.12 (1.17)	
median (min, max)	1.45 (0.83, 2.80)	2.23 (0.18, 5.50)	2.05 (0.18, 5.50)	
house score				<0.001
mean (s.d.)	0.28 (0.11)	0.53 (0.20)	0.47 (0.21)	
median (min, max)	0.31 (0.00, 0.44)	0.50 (0.13, 0.88)	0.44 (0.00, 0.88)	
camp location				0.032
inland	13 (72.2%)	26 (43.3%)	39 (50.0%)	
coastal	5 (27.8%)	34 (56.7%)	39 (50.0%)	
number of siblings				0.186
mean (s.d.)	2.50 (1.86)	3.28 (2.27)	3.10 (2.20)	
median (min, max)	3.00 (0.0, 6.00)	3.00 (0.0, 8.00)	3.00 (0.0, 8.00)	
mean relatedness to camp				0.784
mean (s.d.)	0.13 (0.03)	0.14 (0.09)	0.13 (0.08)	
median (min, max)	0.13 (0.09, 0.19)	0.11 (0.05, 0.40)	0.11 (0.05, 0.40)	
proportion of camp *r* = 0				0.549
mean (s.d.)	0.29 (0.17)	0.33 (0.25)	0.32 (0.23)	
median (min, max)	0.23 (0.12, 0.64)	0.29 (0.00, 0.82)	0.26 (0.00, 0.82)	
proportion of camp *r* > and *r* = 0.25				0.236
mean (s.d.)	0.57 (0.23)	0.49 (0.23)	0.51 (0.23)	
median (min, max)	0.64 (0.09, 0.80)	0.49 (0.03, 0.92)	0.54 (0.03, 0.92)	
proportion of camp *r* > 0.25 and *r* = 0.5				0.401
mean (s.d.)	0.14 (0.08)	0.17 (0.18)	0.17 (0.16)	
median (min, max)	0.09 (0.04, 0.27)	0.12 (0.00, 0.77)	0.12 (0.00, 0.77)	
camp size				0.250
mean (s.d.)	38.33 (11.24)	55 (31.08)	50 (27.15)	
median (min, max)	41 (26, 48)	46 (30, 119)	43.50 (26, 119)	

### (a) Limitations

The major limitation of this study is similar to other observational studies in hunter–gatherers: sample size and study duration. Due to the intensive nature of data collection, we were only able to observe 78 children, each over a 9 h period. Furthermore, given the smaller size of mobile camps, which contained fewer eligible children for the study, our sample only includes 18 children from mobile camps, which has increased the uncertainty about mobile-specific trends. This effect is apparent when looking at the descriptive statistics divided by camp type, where mobile and settled camps do not differ in terms of food storage and time spent in hunter–gathering, a finding that has been reported in the same population with a larger sample [66]. Finally, while our exploratory findings are in line with a wider literature on gendered changes with sedentism, these research questions should be explored more extensively in a dedicated study to this question to examine these processes in-depth.

## 5. Conclusion

We found little evidence that different livelihood strategies associated with mobile and settled camps mapped onto specific behavioural adaptations to buffer risk. There was no evidence in wealthier, settled camps—as a proxy for risk-retention strategies—that mothers' social networks were smaller, or more nuclearized. Nor was there any evidence in resource-poorer mobile camps—as a proxy for risk-pooling strategies—that mothers' social networks were larger, or that non-household individuals were more important allomothers. Our results nonetheless pointed to gender-specific changes occurring alongside sedentism, supporting findings in the wider literature on gender roles in transitioning societies. As camps became more settled girls' childcare and household tasks increased, while boys, being out of camp more, decreased investment in childcare. Our results point to the importance of shifting demographics in larger, more settled camps as playgroups' composition change and grandmothers have fewer dependents, increasing their availability as allomothers. Who provides childcare is dependent on who is present and their wider obligations, features arguably impacted by demography. Overall, while the ‘settled’ and ‘mobile’ Agta communities are part of the same population with a continuous gradient of livelihood change, the flexibility of human childcare is noteworthy, as the identity of the carers did vary. This highlights the flexibility of women's social networks documented here and elsewhere, and demonstrates them as the outcome of demographic trends, gender roles, post-martial residence norms [85] and different modes of subsistence [1,10,56], rather than being constrained. Future research should keep probing the structure of this flexibility to help us better understand the functions of women's social networks and allomothering and how they are expected to change within transitioning populations.

## Data Availability

The code and the data used in these analyses can be found on OSF: https://osf.io/5cghy [120]. The data are provided in the electronic supplementary material [121].
